# High CXCR4 expression impairs rituximab response and the prognosis of R-CHOP-treated diffuse large B-cell lymphoma patients

**DOI:** 10.18632/oncotarget.26588

**Published:** 2019-01-22

**Authors:** Maria Bach Laursen, Linn Reinholdt, Anna Amanda Schönherz, Hanne Due, Ditte Starberg Jespersen, Lykke Grubach, Marianne Schmidt Ettrup, Rasmus Røge, Steffen Falgreen, Suzette Sørensen, Julie Støve Bødker, Alexander Schmitz, Hans E. Johnsen, Martin Bøgsted, Karen Dybkær

**Affiliations:** ^1^ Department of Hematology, Aalborg University Hospital, Aalborg, Denmark; ^2^ Centre for Clinical Research, North Denmark Regional Hospital, Hjørring, Denmark; ^3^ Clinical Cancer Research Center, Aalborg University Hospital, Aalborg, Denmark; ^4^ Department of Clinical Medicine, Aalborg University, Aalborg, Denmark; ^5^ Department of Hematopathology, Aalborg University Hospital, Aalborg, Denmark

**Keywords:** CXCR4, rituximab, diffuse large B-cell lymphoma (DLBCL), prognosis, drug sensitivity

## Abstract

Survival of diffuse large B-cell lymphoma (DLBCL) patients has improved by inclusion of rituximab. Refractory/recurrent disease caused by treatment resistance is, however, a major problem. Determinants of rituximab sensitivity are not fully understood, but effect of rituximab are enhanced by antagonizing cell surface receptor CXCR4. In a two-step strategy, we tested the hypothesis that prognostic value of CXCR4 in DLBCL relates to rituximab treatment, due to a hampering effect of CXCR4 on the response of DLBCL cells to rituximab. First, by investigating the prognostic impact of *CXCR4* mRNA expression separately for CHOP (n=181) and R-CHOP (n=233) cohorts and, second, by assessing the interaction between CXCR4 and rituximab in DLBCL cell lines. High *CXCR4* expression level was significantly associated with poor outcome only for R-CHOP-treated patients, independent of IPI score, *CD20* expression, ABC/GCB and B-cell-associated gene signature (BAGS) classifications. s. For responsive cell lines, inverse correlation was observed between rituximab sensitivity and CXCR4 surface expression, rituximab induced upregulation of surface-expressed CXCR4, and growth-inhibitory effect of rituximab increased by plerixafor, supporting negative impact of CXCR4 on rituximab function. In conclusion, CXCR4 is a promising independent prognostic marker for R-CHOP-treated DLBCL patients, possibly due to inverse correlation between CXCR4 expression and rituximab sensitivity.

## INTRODUCTION

Diffuse large B-cell lymphoma (DLBCL) is the most common type of B-cell-derived non-Hodgkin lymphoma [1], with a varying response and long-term outcome following therapy. Addition of the anti-CD20 monoclonal antibody rituximab (R) to the cyclophosphamide, hydroxydaunorubicin, oncovin, and prednisone (CHOP) treatment regimen has improved survival outcome of DLBCL patients significantly [2]. However, refractory and recurrent disease are major clinical problems due to drug-specific molecular resistance in this heterogeneous disease, and patients with early relapse after rituximab-containing first-line therapy have a poor prognosis [3]. Several mechanisms are involved in rituximab-induced depletion of B-cells including induction of apoptosis by direct signaling, antibody-dependent cellular cytotoxicity, and complement-dependent cytotoxicity [4, 5]. The precise mechanisms of action of rituximab and their relative contribution in patients is not fully understood, and determinants of rituximab sensitivity/resistance in the treatment of DLBCL remain unclear.

DLBCL is a very heterogeneous disease. Gene expression profiling studies have reported the presence of at least two histologically indistinguishable molecular subclasses of DLBCL: the germinal center B-cell-like (GCB) subclass, derived from germinal center cells, and the activated B-cell-like (ABC) subclass, whose expression pattern resembles that of B-cells committed to plasmacytic differentiation [6, 7]. The two molecular subclasses differ in clinical presentation, drug response, genetic aberrations, and gene expression [6, 8–10]. In an approach to extend this current cell-of-origin classification, we recently generated a refined DLBCL classification strategy based on more diverse subset-specific B-cell-associated gene signatures (BAGS) from the normal B-cell hierarchy, by combining fluorescence-activated cell sorting and gene expression profiling of normal human tonsil B-cells, i.e. naïve B-cells, centrocytes, centroblasts, memory B-cells, and plasmablasts [11]. Importantly, BAGS subtyping showed prognostic impact independent of ABC/GCB classification and the International Prognostic Index (IPI) scoring system, the clinical tool used to evaluate the prognosis of DLBCL patients. Notably, within GCB-DLBCL, superior prognosis was observed for the cohort classified as centrocyte subtype (CC) compared to centroblast subtype (CB). Interestingly, sorting of normal tonsil centrocytes and centroblasts for the generation of BAGS was based on differential surface expression of chemokine (C-X-C motif) receptor 4 (CXCR4), with centroblasts displaying higher expression than centrocytes [11].

CXCR4 is a G-protein-coupled chemokine receptor expressed on the surface of the majority of hematopoietic cells [12], whereas its ligand chemokine (C-X-C motif) ligand 12 (CXCL12) is normally produced by stromal cells of lymph nodes, liver, and bone marrow [13, 14]. CXCR4 and CXCL12 play a fundamental role in B-cell development, particularly in establishing the complex germinal center architecture of secondary lymphoid organs [15–17]. However, the CXCL12-CXCR4 axis has been linked to tumor proliferation [18], metastasis [13], and stroma-induced protection from anti-cancer treatment [19–21]; and CXCR4 expression has been associated with poor prognosis [22–24]. In Burkitt lymphoma [19, 20, 25] and chronic lymphocytic leukemia (CLL) [20, 21], the effect of rituximab was enhanced when antagonizing CXCR4 and, recently, we reported a synergistic effect when administering the CXCR4 antagonist plerixafor concomitantly with rituximab to DLBCL cells *in vitro* [26]. The association between CXCR4 expression level and rituximab-specific response has, however, not been thoroughly elucidated in DLBCL.

Here, we tested the hypothesis that the prognostic value of CXCR4 in DLBCL relates to rituximab treatment, due to a hampering effect of CXCR4 on the response of DLBCL cells to rituximab. Complement-dependent cytotoxicity is the mechanism in focus in this study since complement has been reported as essential to the therapeutic activity of rituximab in murine lymphoma models [27, 28] and since disruption of CLL-stromal cell interaction by CXCR4 antagonism *in vitro* was demonstrated to increase the efficacy of rituximab-induced complement-dependent cytotoxicity, whereas this was not the case for rituximab-induced antibody-dependent cellular cytotoxicity [21].

## RESULTS

### *CXCR4* expression is an IPI score, ABC/GCB subclass, and *CD20* expression-independent prognostic marker for R-CHOP-treated DLBCL patients

**Figure 1:**
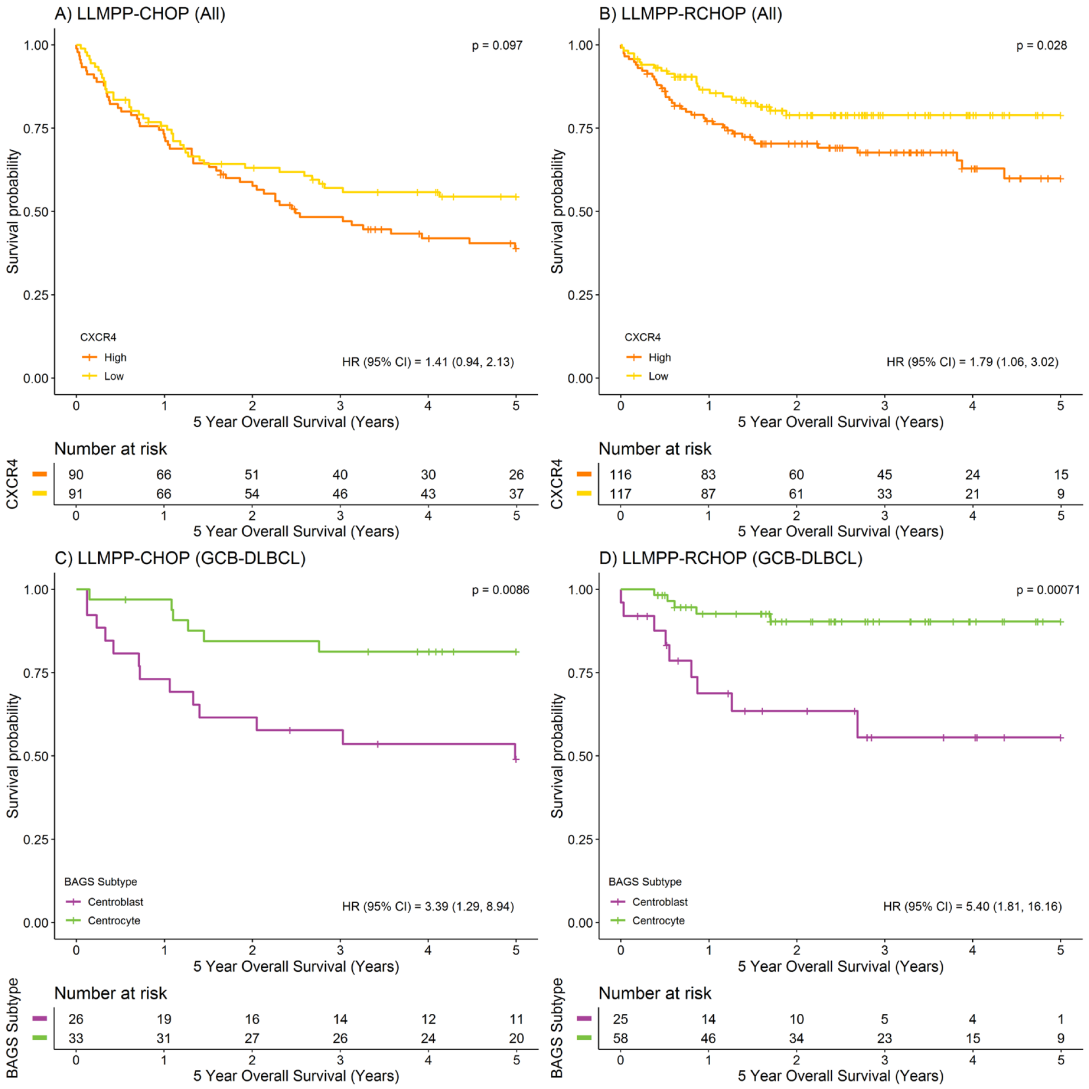
Prognostic value of *CXCR4* expression and BAGS-defined subtypes displaying different levels of *CXCR4* expression **(A-B)** Kaplan-Meier plots depicting 5-year OS for CHOP (n=181) and R-CHOP-treated (n=233) DLBCL patients stratified by *CXCR4* expression level (217028_at), using the median as cut point. **(C-D)** Kaplan-Meier plots depicting 5-year OS for BAGS-defined CC and CB subtypes for CHOP (CC, n=33; CB, n=26) and R-CHOP-treated (CC, n=58; CB, n=25) GCB-DLBCL patients. For comparison of survival curves, the log-rank test was used. For hazard ratio (HR) estimation, a simple Cox’s proportional hazards regression model was used.

To investigate the prognostic value of CXCR4, dichotomized *CXCR4* mRNA expression was analyzed for association to overall survival (OS), in the LLMPP (Lymphoma/Leukemia Molecular Profiling Project) cohort of 414 *de novo* diagnosed DLBCL patients. A strong association between *CXCR4* mRNA expression level and 5-year OS was observed for the R-CHOP-treated DLBCL patient cohort (n=233) but not for the CHOP-treated cohort (n=181), with high *CXCR4* expression characterizing poor outcome (Figure 1A-1B). These observations are in agreement with simple Cox’s proportional hazards regression analyses using *CXCR4* mRNA expression as a continuous variable (Table 1). When performing multiple Cox’s proportional hazards regression analysis, independent variables were only entered into the model if significant results were obtained at the 5% level when performing simple Cox’s proportional hazards regression analyses. Thus, multiple Cox’s proportional hazards regression for the R-CHOP-treated cohort revealed that the prognostic value of *CXCR4* was independent of the already well-established IPI scoring system (Table 1A) and ABC/GCB classification (Table 1B). Since rituximab is an anti-CD20 antibody, it is of particular interest that the prognostic value was also independent of *CD20* expression level (Table 1C). Thus, distinct pathogenetic and prognostic knowledge not already explained by the IPI, ABC/GCB classification or *CD20* expression levels could be captured by the *CXCR4* expression levels.

**Table 1:** *CXCR4* expression is an (A) IPI score, (B) ABC/GCB subclass, (C) *CD20* expression, and (D) GCB-CC/CB subtype-independent prognostic marker for R-CHOP-treated DLBCL patients

A		n^a^	no.	Simple			Multiple		
				HR	95% CI	*P*	HR	95% CI	*P*
CHOP	*CXCR4*	157	77	1.26	0.88-1.80	0.20	-	-	-
	IPI								
	0-1	62	16	1					
	2-3	82	50	3.10	1.76-5.44	8.79E-05	-	-	-
	4-5	13	11	6.45	2.98-13.95	2.18E-06	-	-	-
R-CHOP	*CXCR4*	164	43	1.77	1.04-3.02	0.036	1.77	1.04-3.00	*0.035*
	IPI								
	0-1	70	9	1			1		
	2-3	73	22	2.58	1.19-5.61	0.017	2.69	1.24-5.85	0.013
	4-5	21	12	6.73	2.81-16.14	1.93E-05	6.65	2.78-15.93	2.12E-05

B		n	no.	Simple			Multiple		
				HR	95% CI	*P*	HR	95% CI	*P*
CHOP	*CXCR4*	181	93	1.35	0.96-1.89	0.080	-	-	-
	Subclass								
	ABC	74	49	1					
	GCB	76	28	0.42	0.26-0.66	0.00023	-	-	-
	UC	31	16	0.72	0.41-1.27	0.26	-	-	-
R-CHOP	*CXCR4*	233	60	1.72	1.11-2.66	0.014	1.54	1.01-2.32	*0.042*
	Subclass								
	ABC	93	39	1			1		
	GCB	107	16	0.30	0.17-0.53	4.42E-05	0.32	0.18-0.57	0.00014
	UC	33	5	0.29	0.11-0.73	0.0084	0.28	0.11-0.72	0.0078

C		n	no.	Simple			Multiple		
				HR	95% CI	*P*	HR	95% CI	*P*
CHOP	Entire cohort								
	*CXCR4*	181	93	1.35	0.96-1.89	0.080	-	-	-
	*CD20*	181	93	0.88	0.72-1.06	0.18			
	GCB (CC+CB)^b^								
	*CXCR4*	59	19	1.24	0.53-2.88	0.62	-	-	-
	*CD20*	59	19	0.95	0.54-1.67	0.85	-	-	-
R-CHOP	Entire cohort								
	*CXCR4*	233	60	1.72	1.11-2.66	0.014	1.71	1.12-2.62	*0.014*
	*CD20*	233	60	0.77	0.65-0.91	0.0024	0.75	0.62-0.90	0.0022
	GCB (CC+CB)^b^								
	*CXCR4*	83	14	5.03	1.93-13.09	0.00093	-	-	-
	*CD20*	83	14	0.86	0.49-1.50	0.59	-	-	-

D		n^b^	no.	Simple			Multiple		
				HR	95% CI	*P*	HR	95% CI	*P*
CHOP	*CXCR4*	59	19	1.24	0.53-2.88	0.62	-	-	-
	Subtype								
	GCB-CC	33	6	1					
	GCB-CB	26	13	3.39	1.29-8.94	0.013	-	-	-
R-CHOP	*CXCR4*	83	14	5.03	1.93-13.09	0.00093	4.57	1.71-12.18	*0.0024*
	Subtype								
	GCB-CC	58	5	1			1		
	GCB-CB	25	9	5.40	1.81-16.16	0.0026	4.49	1.48-13.67	0.0081

Connection of *CXCR4* (217028_at) expression level to 5-year OS, analyzed using simple and multiple Cox’s proportional hazards regression models.

^**a**^ IPI score information was not available for all patients, thus cohort sizes are reduced in this setting

^b^ The cohort is restricted to patients classified as GCB-CC or GCB-CB; n, number of samples; no., number of events; HR, hazard ratio; CI, confidence interval; UC, unclassified; -, value is not available since significant results at the 5% level were obtained for only one of the independent variables when performing simple Cox’s proportional hazards regression analysis.

### *CXCR4* expression is a BAGS-defined CC/CB subtype-independent prognostic marker for R-CHOP-treated GCB-DLBCL patients

When evaluating the prognostic impact of BAGS classification separately for ABC and GCB subclasses in a meta-analysis combining information on R-CHOP-treated patients from three individual clinical cohorts (including LLMPP), prognostic stratification was only observed within the GCB cohort, with inferior prognosis for the BAGS-defined CB subtype cohort compared to the CC-classified cohort [11].

**Figure 2:**
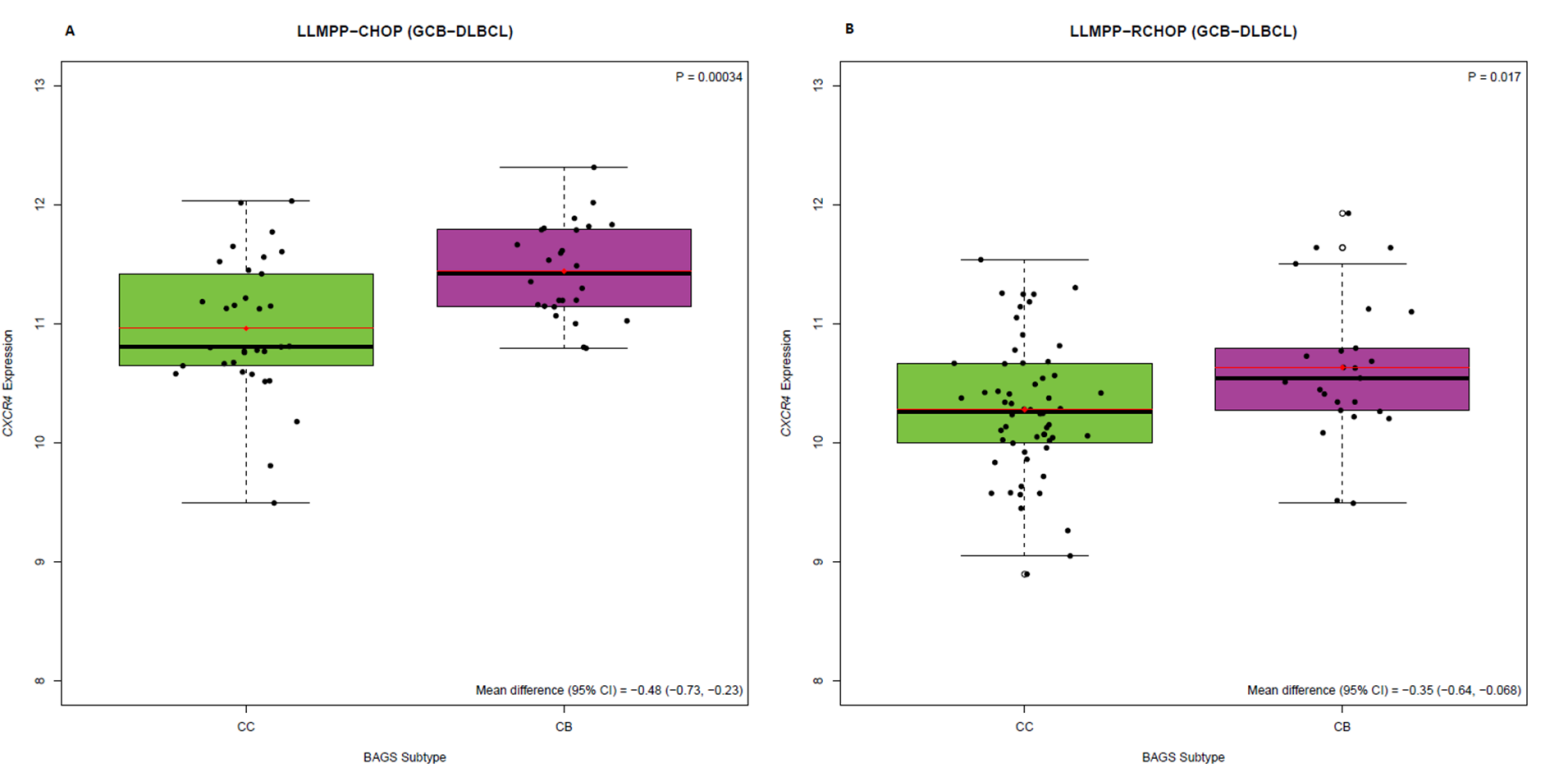
*CXCR4* expression level (217028_at) in BAGS-defined CC and CB subtypes, for GCB-DLBCL patients Individual analysis of the LLMPP **(A)** CHOP treated cohort (GCB-CC, n=33; GCB-CB, n=26) and **(B)** R-CHOP treated cohort (GCB-CC, n=58; GCB-CB, n=25) using Welsch’s *t*-test to test for statistical significance, presenting mean differences with 95% CIs.

Here, we wanted to decipher the role of *CXCR4* expression in this significant difference in outcome. We found that when survival analysis was restricted to the cohort of LLMPP patients classified as GCB subclass and CC (GCB-CC) or CB (GCB-CB) subtype, the CB subtype was still associated with an inferior 5-year OS compared to the CC subtype; regardless of treatment strategy (Figure 1C-1D). The GCB-CB subtype does not seem to benefit much from addition of rituximab since the 3-5-year OS was around 55% for both treatment cohorts, whereas 5-year OS for the GCB-CC cohort increased from approximately 80% to 90% upon addition of rituximab to the treatment regimen. BAGS-defined CC and CB subtypes carry reminiscences of normal centrocyte and centroblast transcriptomic profiles, respectively, and since *CXCR4* expression level is diminished in normal tonsil centrocytes compared to centroblasts [11], we speculated that CXCR4 may hamper the effect of rituximab in the GCB-CB subtype. To determine if this distinct difference in *CXCR4* expression level is sustained in patients, we assessed the *CXCR4* expression level in patient samples and found that the GCB-CB-assigned patient cohort had a higher expression level of *CXCR4* compared to the GCB-CC cohort, whether treated with CHOP or R-CHOP (Figure 2A-2B). To test if *CXCR4* expression level is a surrogate marker for the difference in outcome observed between GCB-CC and GCB-CB-assigned patient cohorts, we used multiple Cox’s proportional hazards regression analysis. We observed *CXCR4* expression to be an independent prognostic marker of 5-year OS following R-CHOP therapy (Table 1D); thus adding prognostic information independent of the BAGS-defined classification. Of notice, the cohort of R-CHOP-treated patients assigned as GCB-CC displayed a significantly higher *CD20* expression level than the GCB-CB cohort (mean difference, 0.59; 95% CI, 0.055-1.1; *P*=0.032); a difference which was not observed for CHOP-treated patients (mean difference, -0.13; 95% CI, -0.57-0.31; *P*=0.56) (Supplementary Figure 1A-1B). When restricting the Cox’s proportional hazards regression analysis to the GCB-CC/GCB-CB cohort, *CXCR4* expression remained a prognostic factor for R-CHOP-treated patients, whereas *CD20* expression level was no longer of significance (Table 1C).

### CXCR4 surface expression level is inversely correlated to the degree of rituximab sensitivity for responsive DLBCL cell lines

To test if the prognostic significance of *CXCR4* mRNA expression can be supported by experimental analyses at the protein level, the relationship between *CXCR4* mRNA and CXCR4 surface expression level was assessed for fourteen drug-naïve human DLBCL cell lines. CXCR4 surface expression was analyzed by flow cytometry and correlated to *CXCR4* gene expression data, documenting a positive correlation (rho=0.92, *P*<2.2e-16) (Supplementary Figure 2A).

**Figure 3:**
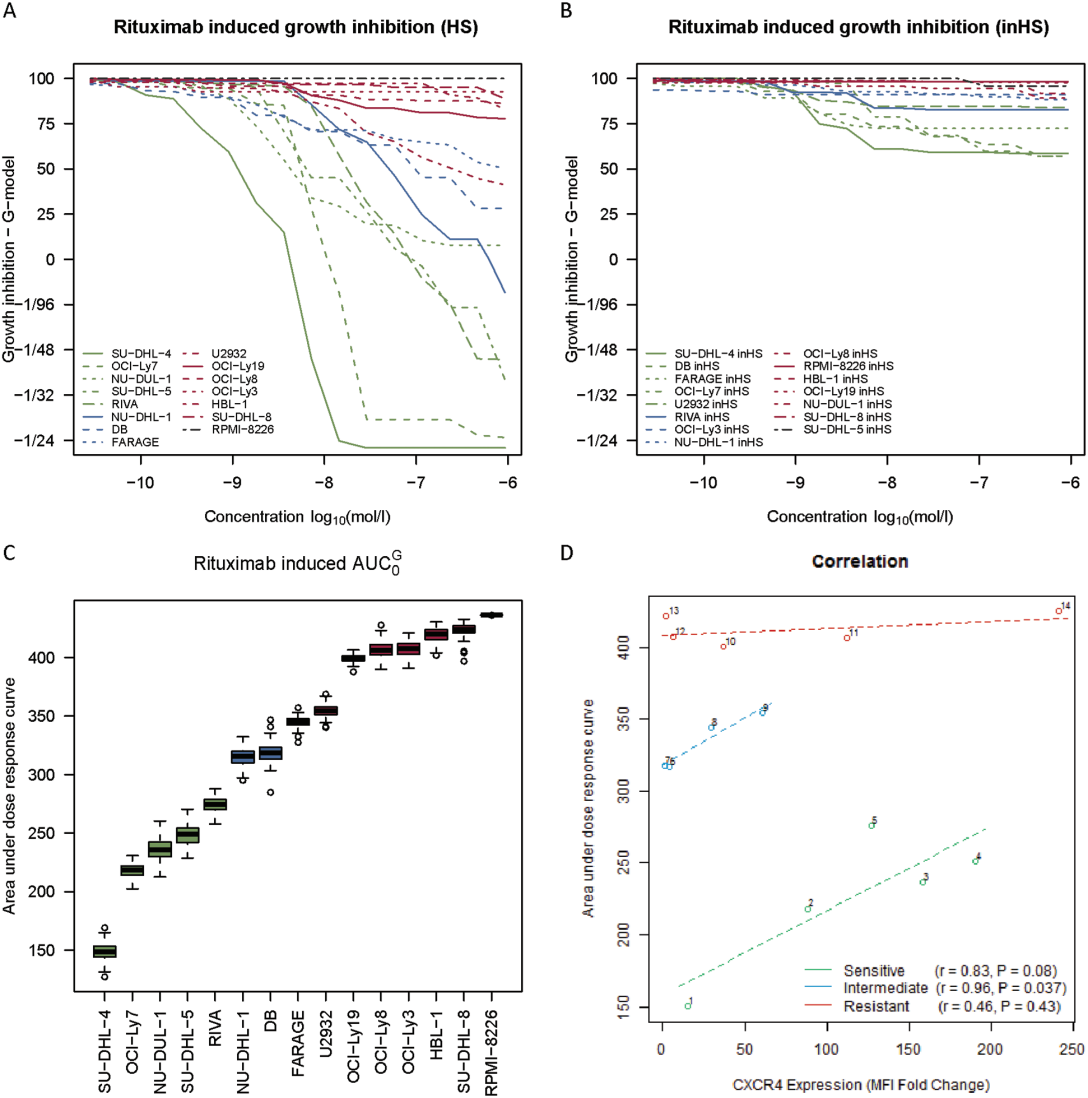
CXCR4 surface expression level is inversely correlated to the degree of rituximab sensitivity for responsive DLBCL cell lines **(A-B)** Systematic rituximab dose-response screens of fourteen human DLBCL cell lines and the CD20-negative multiple myeloma cell line RPMI-8226 (negative control) in a human serum (HS) and heat-inactivated human serum (inHS) setting. Curves are depicted as the average of at least three independent experiments per cell line. **(C)** Box plots illustrating rituximab sensitivity ranking of the cell lines by ${\text{AUC}}_{\text{0}}^{\text{G}}$-values, based on the curves from (A). **(D)** Linear relationship between drug-naïve CXCR4 surface expression level and degree of rituximab sensitivity (${\text{AUC}}_{\text{0}}^{\text{G}}$-values from (C)) for different rituximab response groups, assessed by Pearson’s correlation. CXCR4 surface expression levels are reported as fold changes in median fluorescence intensity (MFI) relative to an unstained control and plotted as the mean of three independent experiments per cell line. Each circle represents a distinct cell line. 1, SU-DHL-4; 2, OCI-Ly7; 3, NU-DUL-1; 4, SU-DHL-5; 5, RIVA; 6, NU-DHL-1; 7, DB; 8, FARAGE; 9, U2932; 10, OCI-Ly19; 11, OCI-Ly8; 12, OCI-Ly3; 13, HBL-1; 14, SU-DHL-8.

The linear relationship between CXCR4 surface expression and the degree of rituximab-induced response was evaluated for the same panel of cell lines. To divide these cell lines into rituximab response groups, systematic dose-response screens were performed (Figure 3A-3B), using MTS-based determination of growth inhibition to measure rituximab-induced effect after 48 hours of drug exposure, applying sixteen decreasing concentrations of rituximab (133.33 μg/ml – 4.07×10^-3^ μg/ml). Two dose-response screens were performed in parallel; one with human serum included as a source of complement (Figure 3A) and one in which the human serum had been heat-inactivated (Figure 3B), illustrating that the drug effect was far more pronounced when complement-dependent cytotoxicity could occur. ${\text{AUC}}_{\text{0}}^{\text{G}}$ (area under dose-response curve obtained by the *G*-model [29])-values were used to assess the degree of rituximab-induced response in the human serum setting (Figure 3C), with a high value corresponding to a low sensitivity towards rituximab and vice versa. Based on these ${\text{AUC}}_{\text{0}}^{\text{G}}$-values, cell lines were divided into tertiles and classified as either rituximab sensitive, intermediate sensitive, or resistant (Figure 3C). For sensitive and intermediate sensitive cell lines, the degree of human serum-dependent rituximab sensitivity was inversely correlated to the level of surface-expressed CXCR4, whereas this was not the case for resistant cell lines (Figure 3D). Notably, even though the resistant cell lines had comparable ${\text{AUC}}_{\text{0}}^{\text{G}}$-values, these cell lines displayed very different CXCR4 surface expression levels. Thus, an increase in CXCR4 surface expression level coincided with decreased rituximab sensitivity for the sensitive and intermediate sensitive cell lines, whereas CXCR4 did not impact the sensitivity status of resistant cell lines. Since rituximab targets the CD20 cell surface receptor, it is relevant to notice that CXCR4 and CD20 surface expression levels did not correlate significantly (sensitive: r=-0.75, *P*=0.14; intermediate sensitive: r=0.65, *P*=0.35; resistant: r=-0.43, *P*=0.48) (Supplementary Figure 2B).

### Rituximab induces upregulation of CXCR4 on the surface of responsive cells in an inactivated human serum setting

**Figure 4:**
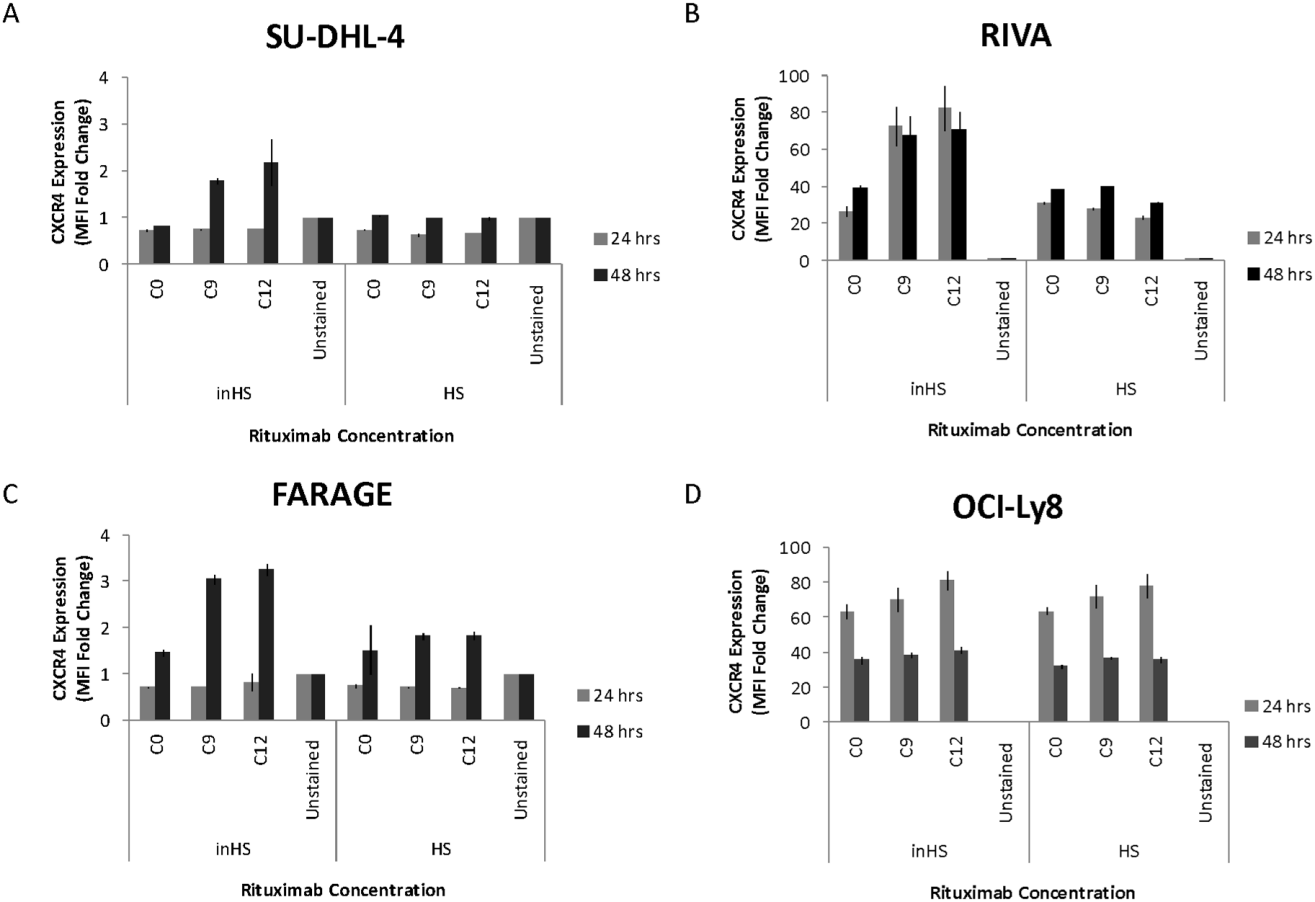
Rituximab induces upregulation of CXCR4 on the surface of responsive cells in an inactivated human serum setting The bar charts show rituximab-induced effect on the CXCR4 surface expression level of **(A-B)** rituximab sensitive, **(C)** intermediate sensitive, and **(D)** resistant DLBCL cell lines, in a human serum (HS) and heat-inactivated human serum (inHS) setting. CXCR4 surface expression levels are reported as fold changes in MFI relative to an unstained control and plotted as the mean of two technical replicates for SU-DHL-4 and three technical replicates for the remaining cell lines. Error bars represent standard deviations. C0, saline; C9, 1.04 μg/ml; C12, 8.33 μg/ml; Unstained, unstained control.

To assess the impact of rituximab on the level of surface-expressed CXCR4, four DLBCL cell lines were exposed to two doses of rituximab (1.04 μg/ml, 8.33 μg/ml) for 24 and 48 hours before flow cytometry-based analysis was conducted (Figure 4). Again, parallel experiments were performed (human serum vs. heat-inactivated human serum). In the rituximab sensitive cell lines SU-DHL-4 (Figure 4A) and RIVA (Figure 4B) and the intermediate sensitive cell line FARAGE (Figure 4C), CXCR4 surface expression level was increased following rituximab exposure, when supplemented with heat-inactivated human serum. In contrast, no increase in CXCR4 surface expression level was observed when rituximab was applied together with human serum. For the resistant cell line OCI-Ly8 (Figure 4D), the impact on expression level was far less than for the responsive cell lines and the effect was not affected by human serum status.

### Antagonizing CXCR4 on the surface of responsive cells increases rituximab efficacy

**Figure 5:**
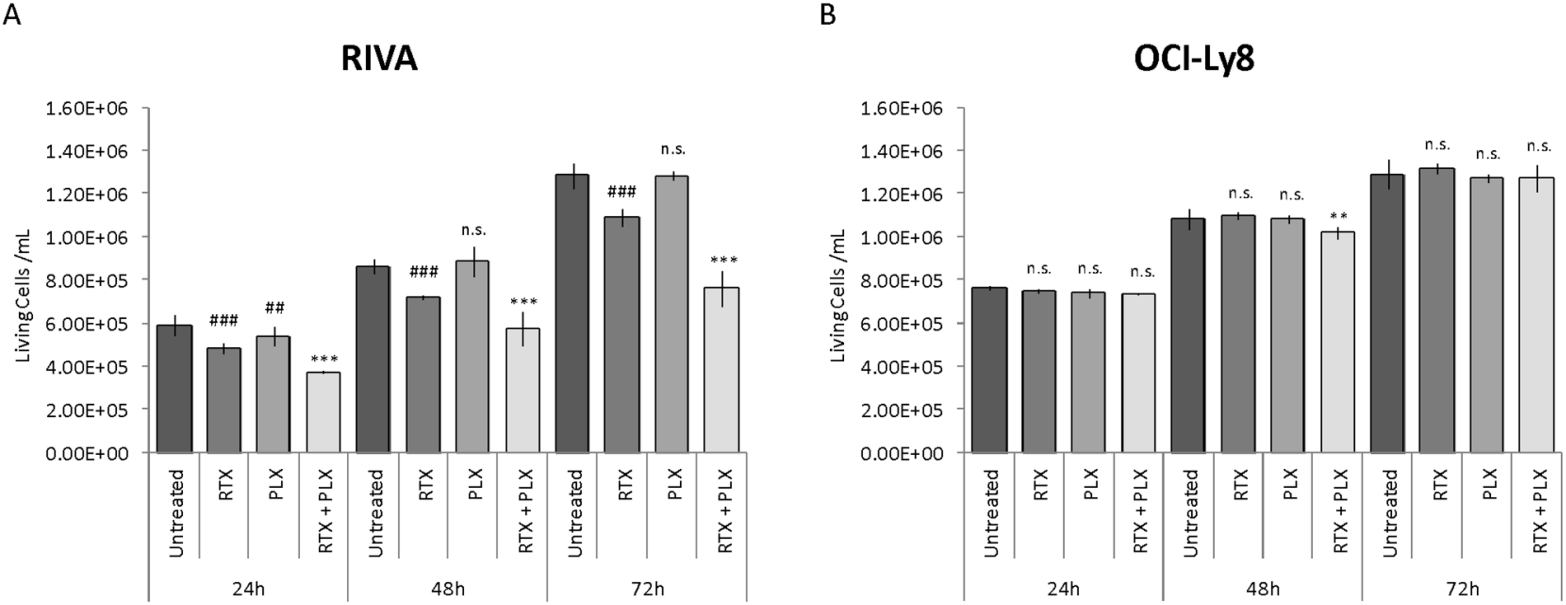
Antagonizing CXCR4 on the surface of responsive cells increases rituximab efficacy The impact of CXCR4 antagonism (plerixafor) on the rituximab-induced response of **(A)** rituximab sensitive and **(B)** resistant DLBCL cell lines. Data are presented as the mean number of living cells/ml of two independent experiments per cell line, with error bars representing standard error of the mean. Linear mixed-effects models were used to assess statistical significance between treatment groups, using untreated control as reference for rituximab and plerixafor monotherapy, and rituximab monotherapy as reference for combination treatment. Untreated, untreated control; RTX, rituximab monotherapy; PLX, plerixafor monotherapy; RTX+PLX, combination treatment; ^##/**^, *P*<0.01; ^###/***^, *P*<0.001; n.s., no significant difference.

The inhibiting effect of CXCR4 on rituximab-induced response in the human serum setting was investigated by combining rituximab treatment with the CXCR4 antagonist plerixafor. RIVA and OCI-Ly8 have comparable CXCR4 surface expression levels (Figure 3D). However, RIVA is sensitive to rituximab whereas OCI-Ly8 is resistant (Figure 3C). Both cell lines were incubated with rituximab (10 μg/ml), plerixafor (500 μM), or rituximab in combination with plerixafor, and the number of living cells/ml was determined by automated cell counting at 24, 48, and 72 hours post-treatment (Figure 5). As expected, we observed rituximab monotherapy to result in a significant decrease in the number of living cells for RIVA (Figure 5A), whereas this was not the case for OCI-Ly8 (Figure 5B). Single agent treatment with plerixafor did not considerably affect the number of living cells, in any of the cell lines. Notably, exposing RIVA cells to a combination of the two drugs increased the effect of rituximab remarkably, whereas only a relatively small decrease in the number of living cells was observed for OCI-Ly8 after 48 hours.

## DISCUSSION

In a two-step strategy, we have tested the hypothesis that the prognostic value of CXCR4 in DLBCL relates to rituximab treatment, due to a hampering effect of CXCR4 on the response of DLBCL cells to rituximab. First, by investigating the prognostic impact of *CXCR4* expression separately for CHOP and R-CHOP cohorts of a clinical DLBCL dataset and, second, by studying the association between CXCR4 surface expression and rituximab sensitivity in a panel of up to fourteen human DLBCL cell lines.

We determined *CXCR4* expression to be significantly associated with outcome in a cohort of 233 R-CHOP-treated DLBCL patients, demonstrating a decreased 5-year OS for patients with high *CXCR4* expression (Figure 1). Several immunohistochemistry-based studies are in agreement [22, 23, 24, 30]. More specifically, high CXCR4 membrane expression was recently associated with disease progression in a small cohort of primary testicular DLBCL patients [23], with poor survival in a cohort of 94 DLBCL patients treated with rituximab-containing regimens (92.5% R-CHOP) [24], and with a tendency towards worse outcome in a cohort of 70 R-CHOP-treated DLBCL patients of which only 8 were classified as CXCR4-negative [30]. Additionally, in a large training/validation cohort of 468/275 *de novo* DLBCL patients, high CXCR4 expression was associated with significantly poorer OS in the overall cohort of R-CHOP-treated DLBCL patients, independent of several pathological and clinical parameters [22]. In our study, multiple Cox’s proportional hazards regression analyses revealed that *CXCR4* expression shows prognostic impact for R-CHOP-treated DLBCL patients, independent of IPI score, ABC/GCB classification, and *CD20* expression. Furthermore, the prognostic impact was independent of our recently reported BAGS-defined CC and CB subtype classification, for GCB-classified patients. These results indicate that distinct pathogenetic and prognostic knowledge not already explained by these prognostic parameters can be captured by *CXCR4* expression level. Combined, this illustrates that CXCR4 expression holds potential as a very useful marker of outcome for R-CHOP-treated DLBCL patientsat the mRNA level. Notably, the prognostic impact of *CXCR4* expression was enhanced when rituximab was included in the chemotherapy regimen, in a manner indicating that the effect of rituximab may be negatively affected by CXCR4. Accordingly, our *in vitro* studies of rituximab-induced response imply that CXCR4 surface expression level correlates inversely with the degree of rituximab response for sensitive and intermediate sensitive, however not for resistant, DLBCL cell lines (Figure 3D). This suggests that CXCR4 is a determinant of rituximab sensitivity degree but is not a controller of intrinsic rituximab resistance mechanisms. Since CXCR4 and CD20 surface expression levels did not correlate significantly, our observations do not merely reflect a difference in CD20 expression level, the target of rituximab.

In our BAGS classification system, CXCR4 surface expression was used as a marker for distinguishing between normal human tonsil centrocytes and centroblasts, with centroblasts displaying higher CXCR4 expression than centrocytes; a pattern also evident at the mRNA expression level [11]. The BAGS-defined CC/CB subtypes are reminiscent of normal centrocytes and centroblasts, respectively, with the GCB-CB-assigned patient cohort displaying higher *CXCR4* expression than the GCB-CC cohort. Importantly, the GCB-CC cohort had a superior 5-year OS compared to the GCB-CB cohort, and the GCB-CC subtype seemed to benefit from addition of rituximab to the treatment regimen, whereas the GCB-CB subtype did not. This suggests that the improved outcome observed for the GCB-CC cohort in the R-CHOP setting may result from a rituximab-dependent mechanism which is hampered by CXCR4 in the GCB-CB subtype. Thus, the difference in *CXCR4* expression level between GCB-CC and GCB-CB patient cohorts could be an explanatory factor for the difference in outcome. Notably, *CXCR4* expression was of significant prognostic value for R-CHOP-treated patients, independent of GCB-CC/GCB-CB classification. Since rituximab is an anti-CD20 antibody, it is relevant to notice that the GCB-CC-assigned R-CHOP-treated patient cohort displayed a higher *CD20* expression level than the GCB-CB cohort. Thus, in addition to the lower *CXCR4* expression level, the higher *CD20* expression level observed for this cohort could be an explanatory factor for the superior prognosis observed for the GCB-CC cohort. In the overall LLMPP R-CHOP cohort, *CD20* and *CXCR4* expression showed significant and independent prognostic impact. In the GCB-CC/GCB-CB-restricted R-CHOP cohort, *CXCR4* expression remained of significant value whereas *CD20* expression did not. Therefore, we propose that *CXCR4* expression is a superior prognostic marker in this defined cohort of DLBCL patients compared to *CD20* expression. However, the reduction in cohort size from 233 to 83 patients might have influenced these results.

In a cohort of 20 non-Hodgkin lymphoma patients, a significantly better prognosis and favorable treatment response was observed for patients experiencing a post-treatment decrease in *CXCR4* expression in the bone marrow [31]. In the light of this finding, it is of particular interest that we demonstrated rituximab to induce upregulation of CXCR4 expression on the surface of responsive cells; especially when combined with our observation that their level of surface-expressed CXCR4 correlated inversely with the degree of rituximab response. Hence, exposure to rituximab might render responsive cells more resistant to rituximab as a consequence of a rituximab-induced upregulation of surface-expressed CXCR4; a mechanism not observed for resistant cells. However, exposure to different physiologic conditions might be required for this to occur, as rituximab-induced CXCR4 upregulation was observed only in the presence of inactivated human serum, whereas correlation of CXCR4 level and rituximab response was demonstrated in a human serum setting. A physiologic *in vivo* evaluation of this proposed negative feedback mechanism is, therefore, warranted. Treatment-induced upregulation of CXCR4 has been reported in other studies. In rectal carcinoma cells, upregulation of CXCR4 was observed following anti-VEGF antibody treatment [32] and, recently, rituximab was shown to induce upregulation in Burkitt lymphoma cell lines and primary DLBCL cells [19]. Metastasis and subsequent acquired rituximab resistance due to stroma-induced protection might be another implication of rituximab-induced CXCR4 upregulation. CXCL12 is normally produced by stromal cells of lymph nodes, liver, and bone marrow [13, 14], creating a CXCL12 concentration gradient that promotes migration of CXCR4-positive normal and malignant hematopoietic cells to these areas. Microenvironment-induced inhibition of drug efficacy has been described in CLL [21] and Burkitt lymphoma [19, 20], where host stromal tissue was shown to protect against rituximab-induced cytotoxicity. Antagonizing CXCR4 abrogated this protective effect, emphasizing the role of CXCR4 in microenvironment-induced rituximab resistance.

CXCR4 surface expression might more directly counteract the effect of rituximab. By comparing the effect of rituximab in the presence and absence of the CXCR4 antagonist plerixafor, we found that the *in vitro* effect of rituximab was enhanced when CXCR4 was antagonized; supporting that CXCR4 impairs the function of rituximab. We observed that cells with comparable CXCR4 surface expression levels but different degrees of sensitivity towards rituximab exhibited different responses to combination treatment, with the effect being remarkably more pronounced for responsive cells, further implying that CXCR4 is involved in rituximab sensitivity but not in intrinsic resistance mechanisms. In agreement with our observations, inhibition of the CXCL12-CXCR4 axis by CXCR4 antagonists improved the efficacy of rituximab in Burkitt lymphoma [19, 20, 25] and CLL [20, 21] and, very recently, we reported that synergistic effect can be assumed when concomitantly administering rituximab and plerixafor to DLBCL cells, with combination treatment effect depending on factors such as drug concentration and administration sequence [26]. Upon binding of CXCL12 to CXCR4, divergent cell signaling pathways are triggered [33], leading to activated PI3K/Akt and MAPK pathways [34, 35]. Interestingly, rituximab efficacy has been shown to depend on the degree of PI3K/Akt and MAPK signaling deregulation [36–38]. Thus, CXCR4-induced deregulation of these pathways might be an explanatory factor for its hampering effect on rituximab response observed for rituximab-responsive DLBCL cells.

Although *in vitro* experiments are useful for studying isolated events in a controlled and very reproducible setting, we acknowledge that *in vitro* experiments cannot capture the complexity of the biological system. Especially factors such as interaction between different mechanisms, exact microenvironmental conditions, and system dynamics are difficult to model. Thus, although disruption of tumor-stromal cell interaction by CXCR4 antagonism did not increase the efficacy of rituximab-induced antibody-dependent cellular cytotoxicity *in vitro* [21], this might be the case *in vivo*. In support of this, neutrophil depletion was demonstrated to abolish plerixafor-induced enhancement of rituximab efficacy in a murine lymphoma model, probably due to the ability of plerixafor to not only disrupt the interaction between tumor and stromal cells but also to mobilize effector cells to the blood [25]. Therefore, it would be interesting to extend the findings of this study by investigating the impact of CXCR4 on mechanisms involved in rituximab-induced depletion of tumor cells in an *in vivo* model system.

In summary, high *CXCR4* expression was significantly associated with poor prognosis for DLBCL patients when rituximab was included in the CHOP treatment regimen. Importantly, this prognostic value of *CXCR4* was independent of IPI score, ABC/GCB classification, *CD20* expression and, for GCB-DLBCL patients, the CC and CB subtypes of our recently defined BAGS classification system. Furthermore, rituximab-induced response was hampered by CXCR4 on the surface of DLBCL cells, with inverse correlation between CXCR4 surface expression level and degree of rituximab sensitivity, for rituximab responsive but not for resistant cell lines; implying that CXCR4 plays a role in rituximab sensitivity but not in intrinsic resistance. Combined, this suggests that CXCR4 holds promise as an independent prognostic marker for R-CHOP-treated DLBCL patients due to a hampering effect of CXCR4 on rituximab-induced response. To further establish this concept, it would be particularly interesting to validate our findings in a prospective study and to explore the biological mechanisms that underlie the proposed inverse relationship between CXCR4 expression and rituximab sensitivity.

## MATERIALS AND METHODS

### Clinical dataset

For clinical data analysis, gene expression data from Affymetrix GeneChip Human Genome U133 Plus 2.0 Array-based analyses of 414 DLBCL patient samples collected prior to treatment initiation (CHOP, n=181; R-CHOP, n=233) were used. The dataset is deposited in the Gene Expression Omnibus repository under accession number GSE10846 and is referred to as the LLMPP cohort. Information about IPI is available (CHOP, n=157; R-CHOP, n=164). Details can be found in Lenz *et al.* [10].

### Proof of principle validation of microarray based CXCR4 expression by quantitative digital droplet PCR

To ensure that microarray based determination of CXCR4 expression is trustworthy we performed a small validation assessment on an in house cohort of 52 DLBCL de novo clinical diagnostic samples (GSE110376) for correlation of CXCR4 probe 217028_at expression on GeneChip Human Genome U133 Plus 2.0 and relative expression of CXCR4 determined by digital droplet PCR (TaqMan #Hs00237052_m1) (Supplementary Figure 3). Person correlation coefficient was 0.84 documenting a fair reproducibility across detection methods despite differences in time; e.g. microarrays were run in 2015 whereas ddPCR were performed in 2018 using remaining RNA aliquots stored at -80 °C and normalization procedures where RMA was used for microarrays vs usage of similar RNA-equivalent cDNA input in ddPCR normalized against mean of reference genes TBP and PPIA.

### Human DLBCL cell lines

Fourteen human DLBCL-derived cell lines, i.e. SU-DHL-4, OCI-Ly7, NU-DUL-1, SU-DHL-5, RIVA, NU-DHL-1, DB, FARAGE, U2932, OCI-Ly19, OCI-Ly8, OCI-Ly3, HBL-1, and SU-DHL-8, were included (Table 2). The CD20-negative multiple myeloma cell line, RPMI-8226, was included as negative control. Cells were maintained under appropriate culturing conditions, and their identity was validated regularly using the DNeasy Blood and Tissue Kit (Qiagen, Copenhagen, Denmark), the AmpFISTR^®^ Identifiler^®^ PCR Amplification Kit (Applied Biosystems, CA, USA), and capillary electrophoresis (Eurofins Medigenomix GmbH, Applied Genetics, Germany). A unique identification was generated by use of the Osiris program and the German Collection of Microorganisms and Cell Cultures (DSMZ) database (http://www.dsmz.de/services/services-human-and-animal-cell-lines/online-str-analysis.html), checking the length of nine out of sixteen short tandem repeats. The EZ-PCR Mycoplasma Test Kit (Biological Industries, Beit HaEmek, Israel) was used to exclude mycoplasma-induced changes. Whenever incubation details are not supplied, cells were incubated in a humidified atmosphere at 37°C, 5% CO_2_.

**Table 2:** Cell line specifications

Cell Line	Cell Type	Supplier/Purchase Information	Culture Medium^a^	Seeding Concentration (×10^6^ cells/ml)	Rituximab Response^b^
SU-DHL-4	DLBCL	DSMZ Acc. 495	RPMI 1640 + 10% FBS	0.3	Sensitive
OCI-Ly7	DLBCL	1	RPMI 1640 + 10% FBS	0.3	Sensitive
NU-DUL-1	DLBCL	DSMZ Acc. 579	RPMI 1640 + 15% FBS	0.3	Sensitive
SU-DHL-5	DLBCL	DSMZ Acc. 571	RPMI 1640 + 20% FBS	0.3	Sensitive
RIVA	DLBCL	1	RPMI 1640 + 10% FBS	0.3	Sensitive
NU-DHL-1	DLBCL	DSMZ Acc. 583	RPMI 1640 + 10% FBS	0.15	Intermediate
DB	DLBCL	DSMZ Acc. 539	RPMI 1640 + 20% FBS	0.15	Intermediate
FARAGE	DLBCL	1	RPMI 1640 +10% FBS	0.3	Intermediate
U2932	DLBCL	1	RPMI 1640 + 10% FBS	0.5	Intermediate
OCI-Ly19	DLBCL	1	RPMI 1640 + 10% FBS	0.3	Resistant
OCI-Ly8	DLBCL	2	RPMI 1640 + 10% FBS	0.3	Resistant
OCI-Ly3	DLBCL	1	IMDM + 20% inHS + 55μM 2-mercaptoethanol	0.15	Resistant
HBL-1	DLBCL	1	RPMI 1640 + 10% FBS	0.3	Resistant
SU-DHL-8	DLBCL	1	RPMI 1640 + 10% FBS + 2mM L-glutamin	0.3	Resistant
RPMI-8226	Multiple myeloma	DSMZ Acc. 402	RPMI 1640 + 10% FBS	0.15	Negative control

^a^ All culture media were supplemented with 1% penicillin-streptomycin

^b^ Cell lines are ranked as rituximab sensitive, intermediate sensitive, or resistant according to their ${\text{AUC}}_{\text{0}}^{\text{G}}$-value in the human serum setting; 1, Kind gift from Associate Professor Jose A. Martinez-Climent, MD, PhD, University of Navarra, Pamplona, Spain; 2, Kind gift from Professor Hans Messner, MD, PhD, University of Toronto, Toronto, Canada; FBS, fetal bovine serum; inHS, heat-inactivated human serum; DSMZ, Deutsche Sammlung von Mikroorganismen und Zellkulturen GmbH; RPMI, Roswell Park Memorial Institute; IMDM, Iscove's Modified Dulbecco's Medium.

### MTS-based dose-response screens

Systematic rituximab dose-response screens were performed for all cell lines. Cells were seeded in 96-well culture plates at concentrations ranging from 0.15-0.5×10^6^ cells/ml (Table 2) and incubated overnight before applying saline or sixteen decreasing concentrations of rituximab (MabThera^®^, Roche, Copenhagen, Denmark) in 2-fold dilutions from C16=133.33 μg/ml to C1=4.07×10^-3^ μg/ml. After 30 minutes of incubation, 20% human serum (Pooled Human AB Serum, Novakemi AB, Handen, Sweden) or heat-inactivated human serum was added, to enable assessment of rituximab-induced complement-dependent cytotoxicity [4]. Impact of rituximab was tested by determining the number of metabolically active cells, using an MTS-based colorimetric method. An MTS-containing reagent (CellTiter 96 Aqueous One Solution Reagent, Promega, WI, USA) was added immediately and 48 hours after serum addition. Following incubation for exactly 2 hours, absorbance at 492 nM was measured using an Optima-Fluostar (BMG LABTECH, Ortenberg, Germany). Cell count estimates were obtained at approximately 1 and 49 hours since these time points represent the center of MTS exposure. Only non-border wells were used for subsequent analysis to avoid border effects. To achieve high reproducibility, technical triplicates were included, and the entire experiment was repeated at least thrice per cell line.

### Flow cytometry

Drug-naïve CXCR4 surface expression was analyzed for all cell lines. Approximately 1×10^6^ cells were suspended in 40 μl Stain Buffer (phosphate-buffered saline containing 2% fetal bovine serum), adding 10 μl PE-conjugated anti-CXCR4 antibody (clone 12G5, Beckman Coulter, Copenhagen, Denmark). Following incubation (15 min, room temperature, in dark), cells were washed with 3 ml Stain Buffer before centrifugation (500×*g*, 5 min, room temperature) and resuspension in 0.4 ml Stain Buffer. Three independent experiments were conducted per cell line.

CXCR4 surface expression upon rituximab exposure was analyzed for SU-DHL-4, RIVA, FARAGE, and OCI-Ly8. Cells were seeded (0.3×10^6^ cells/ml) in 24-well culture plates and incubated overnight. After addition of saline or rituximab (C9=1.04 μg/ml, C12=8.33 μg/ml), cells were incubated for 30 minutes, after which either 20% human serum or heat-inactivated human serum was added. Following incubation for 24 and 48 hours, cells were harvested, washed with 2 ml phosphate-buffered saline, resuspended in 80 μl Stain Buffer, and stained by adding 20 μl PE-conjugated anti-CXCR4 antibody (clone 12G5, Beckman Coulter, Copenhagen, Denmark). Following incubation (15 min, room temperature, in dark), cells were washed with 3 ml Stain Buffer before centrifugation (500×*g*, 5 min, room temperature) and resuspension in 0.25 ml Stain Buffer. At least two technical replicates were included.

For all experiments, unstained controls were included to detect auto-fluorescence, a BD FACSCanto™ II (BD Biosciences, Copenhagen, Denmark) flow cytometer was used for acquisition, and FlowJo Software (Tree Star Inc., OR) for data analysis.

### Cell counting

Enumeration of living cells (RIVA, OCI-Ly8) was performed by automated cell counting. Cells were seeded (0.3×10^6^ cells/ml) in 24-well culture plates and incubated overnight before drug/saline and 20% human serum was added. Rituximab (10 μg/mL) and plerixafor (500 μM) (InSolution™ CXCR4 Antagonist I, AMD3100, Merck Millipore, Copenhagen, Denmark) was added concomitantly or individually. Following 24, 48, and 72 hours of drug exposure, cells were counted using a NucleoCounter^®^ NC-200™ (ChemoMetec, Copenhagen, Denmark) automated cell counter. Three separate countings were performed per well, and experiments were repeated twice.

### Statistical analysis

For survival analysis, the hybridization-specific *CXCR4* probe set 217028_at was chosen for assessment of expression level, which was either included as a dichotomized (median split) or continuous variable. Survival analysis was performed using Kaplan-Meier analysis, log-rank test, and simple Cox’s proportional hazards regression analysis. Multiple Cox’s proportional hazards regression analysis was used to adjust for either IPI score, ABC/GCB subclass, *CD20* expression, or BAGS-defined subtype [11]. To test for difference in mean expression level between subtypes, Welch’s *t*-test was used.

For rituximab dose-response screens, raw absorbance values were pre-processed using a previously described model-based procedure [29]. Dose-response curves and time-independent summary statistics (${\text{AUC}}_{\text{0}}^{\text{G}}$-values) were estimated using the *G*-model combined with an established statistical analysis workflow [29]. Pearson’s correlation coefficients were estimated to determine the strength of the linear relationship between expression levels and ${\text{AUC}}_{\text{0}}^{\text{G}}$-values for each rituximab response group, whereas linear models which take grouping according to rituximab sensitivity into account were used for plotting lines of best fit, and linear mixed-effects models applied to evaluate the effect of treatment on the number of living cells, with experimental replicate as random effect.

Statistical analyses were performed using the statistical software R [39], and *P*<0.05 was considered statistically significant.

## SUPPLEMENTARY MATERIALS FIGURES



## Abbreviations

ABC: activated B-cell-like
${\text{AUC}}_{\text{0}}^{\text{G}}$: area under dose-response curve obtained by the *G*-model
BAGS: B-cell-associated gene signature
CB: centroblast subtype
CC: centrocyte subtype
CHOP: cyclophosphamide, hydroxydaunorubicin, oncovin, and prednisone
CXCL12: chemokine (C-X-C motif) ligand 12
CXCR4: chemokine (C-X-C motif) receptor 4
DLBCL: diffuse large B-cell lymphoma
DSMZ: German Collection of Microorganisms and Cell Cultures
GCB: germinal center B-cell-like
GCB-CB: GCB subclass and CB subtype
GCB-CC: GCB subclass and CC subtype
IPI: International Prognostic Index
LLMPP: Lymphoma/Leukemia Molecular Profiling Project
OS: overall survival
R-CHOP: rituximab + CHOP
