# Supplementary material for: High CXCR4 expression impairs rituximab response and the prognosis of R-CHOP-treated diffuse large B-cell lymphoma patients
Source: Oncotarget. 2019 Jan 22;10(7):717–31. doi: 10.18632/oncotarget.26588 (PMC6366826; doi:10.18632/oncotarget.26588)
Supplement: Supplementary file 1 [file oncotarget-10-717-s001.pdf]

High CXCR4 expression impairs rituximab response and the prognosis of R-CHOP-treated diffuse large B-cell lymphoma patients

SUPPLEMENTARY MATERIALS

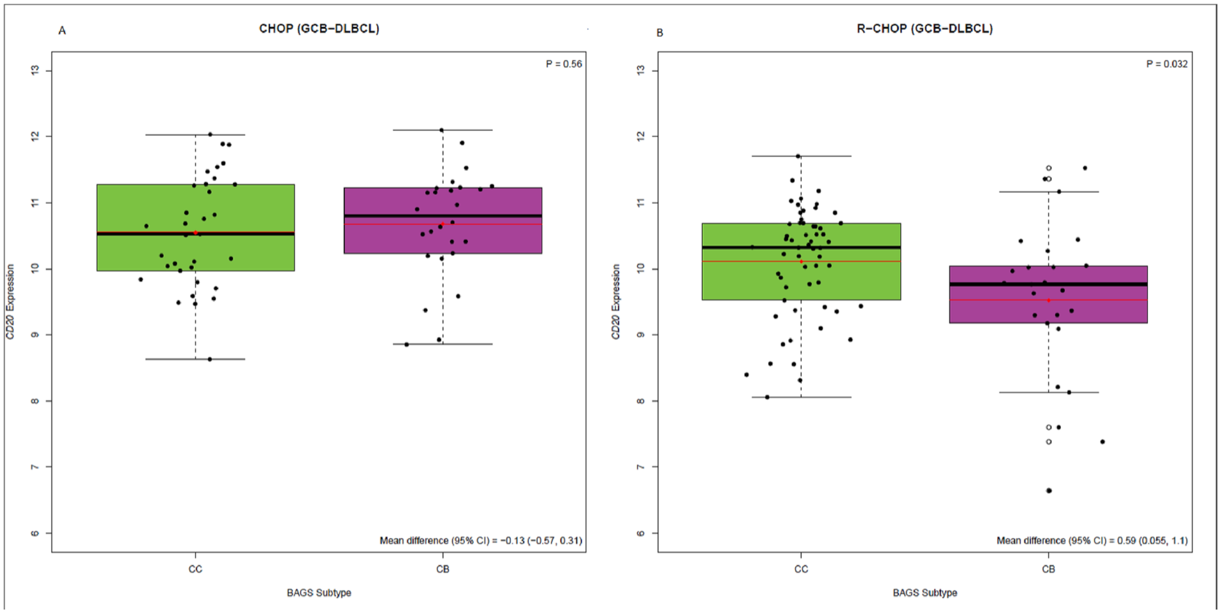

Supplementary Figure 1: *CD20* expression level is higher in the BAGS-defined CC subtype cohort than in the CB cohort, for R-CHOP-treated GCB-DLBCL patients. *CD20* mRNA expression levels (228599_at) were previously determined by microarray-based analysis (deposited in the Gene Expression Omnibus repository under accession number GSE53798). Comparison of *CD20* expression level (228599_at) between BAGS-defined CC and CB subtypes for **(A)** CHOP (CC, n=33; CB, n=26) and **(B)** R-CHOP-treated (CC, n=58; CB, n=25) GCB-DLBCL patients. Welch's *t*-test was used to test for difference in expression level between subtypes.

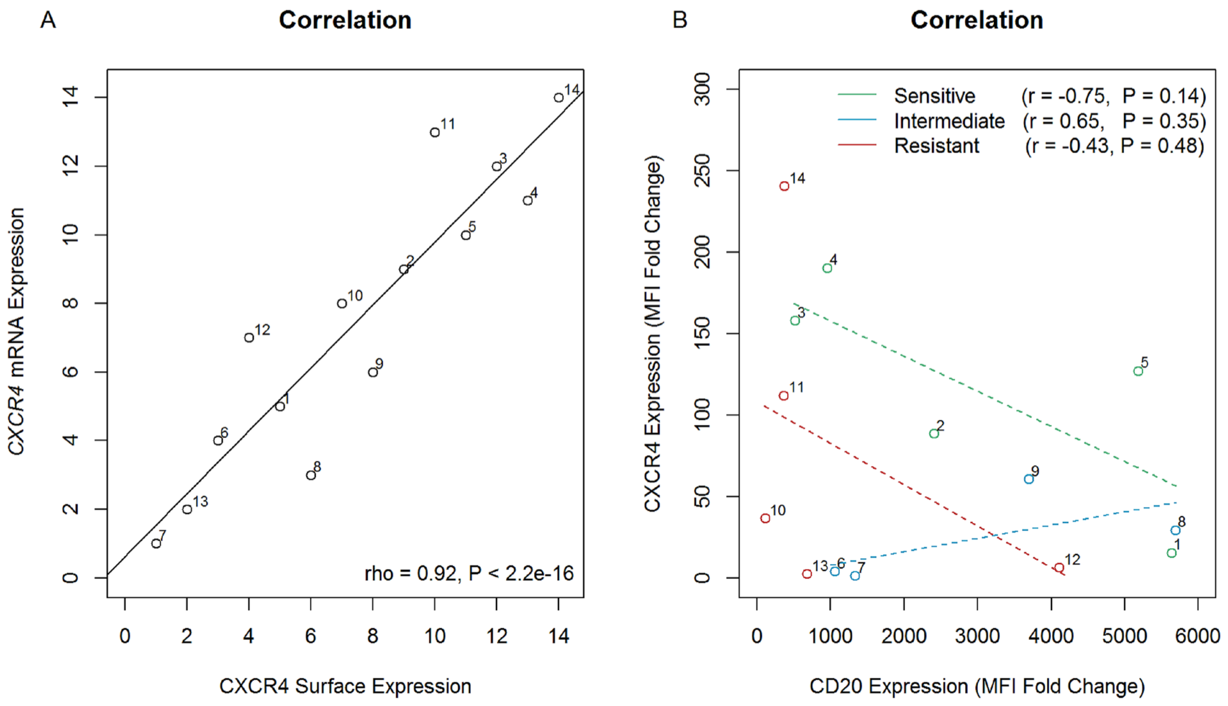

Supplementary Figure 2: Additional correlation analyses related to CXCR4 surface expression of drug-naïve DLBCL cell lines. (A) Degree of association between levels of surface-expressed CXCR4 and *CXCR4* mRNA expression was assessed using Spearman's correlation analysis. **(B)** Linear relationship between CXCR4 and CD20 surface expression levels for different rituximab response groups, assessed by Pearson's correlation. Surface expression levels are reported as fold changes in MFI relative to an unstained control and plotted as the mean of at least two independent experiments per cell line. MFI-values were determined by flow cytometry-based analysis of fourteen drug-naïve human DLBCL cell lines, using PE-conjugated anti-CXCR4 antibody or APC-H7-conjugated anti-CD20 antibody for staining. *CXCR4* mRNA expression levels (217028_at) were previously determined by microarray-based analysis (deposited in the Gene Expression Omnibus repository under accession number GSE53798). Each circle represents a distinct cell line. 1, SU-DHL-4; 2, OCI-Ly7; 3, NU-DUL-1; 4, SU-DHL-5; 5, RIVA; 6, NU-DHL-1; 7, DB; 8, FARAGE; 9, U2932; 10, OCI-Ly19; 11, OCI-Ly8; 12, OCI-Ly3; 13, HBL-1; 14, SU-DHL-8.

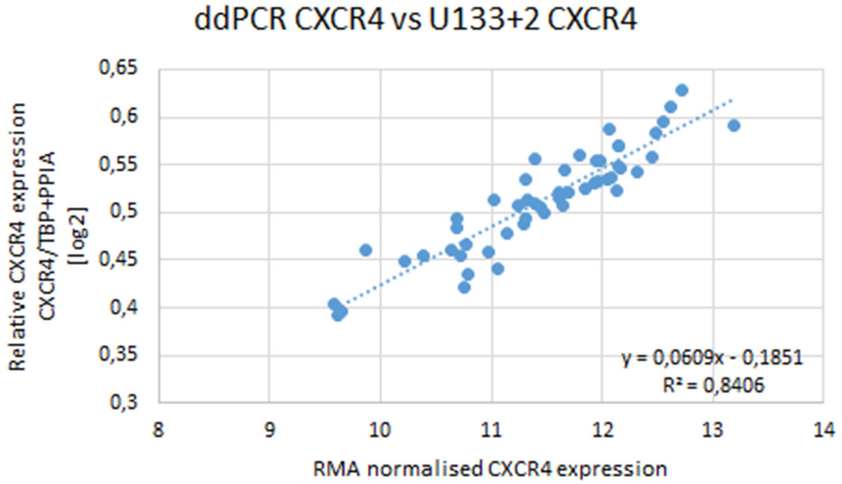

Supplementary Figure 3: *CXCR4* expression detected by microarray validated by ddPCR. An in house cohort of 52 DLBCL samples (GSE110376) were tested for correlation of *CXCR4* mRNA expression levels (217028_at) determined by microarray-based analysis (U133+2) and relative expression of *CXCR4* determined by digital droplet PCR (ddPCR) using CXCR4 (Y-axis) documenting a fair correlation (r^2=0.84) between the two platforms.
